# Antimicrobial Peptide CPP-C3M4 Attenuates *Salmonella*-Induced Liver Inflammation and Oxidative Stress in Lambs

**DOI:** 10.3390/ani16152290

**Published:** 2026-07-23

**Authors:** Chunyuan Pan, Wenhao He, Wanxin Tian, Wanxin Xu, Hongyan Li, Chenxue Zhang, Sijia Liu, Xiaodong Xu, Yumeng Qin, Aizhong Zhang, Ning Jiang

**Affiliations:** 1 Key Laboratory of Feed Resource Efficient Utilization and Nutrition Manipulation in Cold Region of Heilongjiang Province, College of Animal Science and Veterinary Medicine, Heilongjiang Bayi Agricultural University, Daqing 163319, China; pancy210@byau.edu.cn (C.P.); 15546358938@163.com (W.H.); twanxinnn@163.com (W.T.); 18745743782@163.com (W.X.); lhy_student@163.com (H.L.); 18345527598@163.com (S.L.); 15776507853@163.com (X.X.); 18045700109@163.com (Y.Q.); 2 College of Veterinary Medicine, Inner Mongolia Agricultural University, Hohhot 010018, China; x408337660@163.com; 3 Daqing Agricultural and Rural Social Service Center, Daqing 163000, China; 4 Longjiang Wagyu Biotechnology Co., Ltd., Qiqihar 161100, China; 5 Agriculture and Rural Affairs Bureau of Wangkui County, Suihua 152100, China

**Keywords:** *Salmonella*, liver injury, inflammation, oxidative stress, transcriptomics, lambs

## Abstract

In this study, we investigated the protective effects of CPP-C3M4 against *Salmonella*-induced hepatic injury in lambs. Thirty 72-day-old Hu sheep were randomly divided into five groups: the control group (CON), the *Salmonella*-infected group (ST), and three CPP-C3M4 pretreatment groups at low, medium and high doses (CPP-C3M4-L, CPP-C3M4-M, and CPP-C3M4-H). The results showed that administration of CPP-C3M4 via duodenal fistula significantly alleviated liver enlargement and histopathological damage caused by *Salmonella* infection. CPP-C3M4 improved liver function by reducing alanine aminotransferase (ALT), aspartate aminotransferase (AST), and triglyceride (TG) levels. It enhanced the liver antioxidant capacity by increasing glutathione peroxidase (GSH-Px), catalase (CAT), and superoxide dismutase (SOD) activities. It also alleviated inflammatory responses by downregulating the mRNA expression of interleukin-1β (*IL-1β)*, interleukin-6 (*IL-6*), tumor necrosis factor-α (*TNF-α*), interleukin-2 (*IL-2*), toll-like receptor 2 (*TLR2*), and toll-like receptor 9 (*TLR9*). Transcriptomic Kyoto Encyclopedia of Genes and Genomes (KEGG) analysis further confirmed that CPP-C3M4 exerts its protective effects by suppressing multiple inflammatory pathways, with the interleukin-17 (IL-17) signaling axis being a key target of regulation. These results indicate that CPP-C3M4 is a promising alternative antimicrobial substance for preventing and alleviating *Salmonella*-induced liver injury in lambs.

## Simple Summary

Salmonellosis is an important infectious disease in the sheep industry and poses a serious threat to both pregnant ewes and newborn lambs. Antimicrobial peptides (AMPs) exhibit broad-spectrum antimicrobial activity and good safety profile. They can also improve the production performance and immune function of livestock. The antimicrobial peptide CPP-C3M4 was prepared by conjugating synthetic peptide C3M4 with a cell-penetrating peptide (CPP), which was screened via phage display technology in our laboratory. CPP-C3M4 was recombinantly expressed in *Pichia pastoris*, and processed through fermentation, purification, and spray drying to yield CPP-C3M4 powder. Lambs were infused with CPP-C3M4 powder via duodenal fistula, followed by establishment of a *Salmonella* infection model. Relevant indices and liver transcriptomic analysis were performed to evaluate the protective effects of CPP-C3M4 against *Salmonella*-induced liver injury in lambs. The results showed that pretreatment with different doses of CPP-C3M4 alleviated *Salmonella*-induced histopathological damage, enhanced antioxidant capacity, and regulated the expression of inflammation-related factors in lambs. Transcriptomic analysis revealed the key signaling pathways underlying the hepatoprotective effect of CPP-C3M4, providing a theoretical basis for the application of AMP-based preparations in the prevention and treatment of *Salmonella*-induced hepatic injury.

## 1. Introduction

As a Gram-negative intracellular bacterium, *Salmonella* can invade intestinal epithelial cells [1], secrete virulence factors [2], and trigger host inflammatory responses [3], thereby causing gastroenteritis and septicemia in livestock, particularly in young animals [4]. Among more than 2000 *Salmonella* serotypes, *Salmonella* Typhimurium features a broad host range and considerable pathogenic potential, posing a significant threat to young livestock such as piglets and lambs [5,6]. Traditional antibiotics including quinolones and *β*-lactams have prominent defects against intracellular *Salmonella*, such as poor cell membrane penetration [7], as well as disruption of intestinal flora homeostasis and acceleration of antimicrobial resistance gene transfer [8,9].

Cell-penetrating peptide-conjugated antimicrobial peptides (CPP-AMPs) are promising antibiotic alternatives for controlling intracellular *Salmonella* infections. CPP-AMPs combine the broad-spectrum bactericidal activity of antimicrobial peptides (AMPs) with the membrane-penetrating capability of cell-penetrating peptides (CPPs) [10]. Some AMPs bind negatively charged bacterial membranes via cationic residues to destroy membrane integrity [11]. While CPP moieties mediate endocytosis or direct transmembrane transport to deliver active peptides into host cells for eliminating intracellular *S.* Typhimurium [12,13]. Unlike conventional antibiotics that can induce drug resistance, CPP-AMPs kill bacteria through physical membrane disruption, greatly mitigating the risk of resistance [14]. In addition, some AMPs inhibit excessive pro-inflammatory cytokine release, which may contribute to alleviating inflammatory tissue injury [15].

*Salmonella* infections in sheep can affect all breeds, ages and sexes, with newly weaned lambs being particularly susceptible [16,17]. The disease has been reported to cause considerable economic losses to the sheep industry worldwide. In a documented outbreak of *S.* Typhimurium in a 4000-ewe flock in the UK, approximately 100 ewes and 700 lambs died, with total direct and indirect costs estimated at over £71,000 [18]. In China, the disease is also recognized as a cause of economic losses in sheep production [19,20]. Hu sheep is an important meat-producing sheep breed in China. Its lambs have immature intestinal epithelial barrier function, weak antioxidant defenses, and insufficient interleukin-10 (IL-10)-mediated anti-inflammatory capacity, making them highly susceptible to *S.* Typhimurium colonization [21,22]. *Salmonella* infections in sheep are predominantly associated with diarrheal enteritis, particularly in lambs [23,24,25]. Although this enteric-type form represents the most common clinical presentation, severe cases may progress to systemic infection. *Salmonella* can invade the bloodstream from damaged intestinal mucosa or mesenteric lymph nodes, causing systemic infection with multifocal hepatocellular necrosis and inflammatory cell infiltration in the liver [26,27]. While liver lesions caused by *Salmonella* are not the primary clinical manifestation in sheep, severe liver injury can significantly aggravate the severity of salmonellosis in lamb [28]. Given its severe consequences that this complication can have for affected animals, exploring treatment options for this specific complication remains clinically relevant, and there is a lack of effective interventions that can specifically target intracellular bacteria and alleviate subsequent liver damage.

Against this backdrop, we constructed the cell-penetrating peptide-conjugated antimicrobial peptide CPP-C3M4 and investigated its protective effects against *S.* Typhimurium-induced liver injury in Hu lambs, with the aim of providing a potential strategy for the prevention and treatment of invasive salmonellosis in young sheep.

## 2. Materials and Methods

### 2.1. Experimental Strain

*Salmonella* Typhimurium CMCC50115, purchased from the China Medical Culture Collection (CMCC, Beijing, China), was used as the experimental strain.

### 2.2. Preparation of CPP-C3M4 Powder

CPP-C3M4 was previously constructed and synthesized in our laboratory [29,30], with the amino acid sequence GLLKGLLKKIGKKIRVGVRTRAVLNSLID. To facilitate subsequent detection, this construct was fused with enhanced green fluorescent protein (EGFP) to generate CPP-C3M4-EGFP. The fusion protein was expressed in a recombinant *Pichia pastoris* engineering strain for fermentation. Following centrifugation of the fermentation broth at 5000 rpm (15 min), the supernatant was passed through a 0.45 μm membrane. The filtrate was spray-dried using a YC-2100 spray dryer (Shanghai Pilotech Instrument & Equipment Co., Ltd., Shanghai, China) to obtain antimicrobial peptide powder, with a target protein content of 22.5 mg/g.

### 2.3. Experimental Animals and Experimental Design

Thirty 72-day-old healthy Hu sheep with similar body weights (18.29 ± 0.49 kg) were surgically fitted with permanent duodenal cannulas. They were then randomly assigned into five groups (n = 6 per group; three males and three females): the control group (CON, basal diet only), the *S.* Typhimurium challenge group (ST, basal diet + challenge), and three CPP-C3M4 pretreatment groups (CPP-C3M4-L, CPP-C3M4-M, and CPP-C3M4-H; basal diet + challenge).

The trial consisted of a 7-day initial adaptation phase followed by a 35-day experimental period. During the adaptation period and the 30 preceding days of the experimental period, all lambs were kept in the Experimental Animal Center of the College of Animal Science and Technology, Heilongjiang Bayi Agricultural University. On days 31 to 32 of the experimental period, all lambs were transported to the Experimental Animal Center of Harbin Pharmaceutical Group Biological Vaccine Co., which is equipped with the specialized biosafety containment facilities required for the *Salmonella* challenge tests. The subsequent challenge experiments, slaughter, and sample collection were conducted on these lambs at this facility. The *Salmonella* challenge was performed on days 33 to 35 of the experimental period. At the completion of the experiment, all lambs were humanely euthanized. The experimental animals were first deeply anesthetized using intravenous propofol (at a dose of 4–6 mg/kg body weight, which was gradually adjusted based on the onset of anesthesia, as evidenced by the loss of jaw tone, the disappearance of the eyelid reflex, and the absence of response to noxious stimuli), followed by exsanguination. Death was confirmed by verifying the cessation of the heartbeat, the absence of respiration, and the disappearance of the corneal reflex. The euthanasia procedure followed the AVMA Guidelines for the Euthanasia of Animals (2020 Edition). Tissue samples were collected immediately after death confirmation. The whole process of the animal experiment was evaluated and approved by the Animal Ethics Committee of Heilongjiang Bayi Agricultural University (Approval Code: DWKJXY2022064).

During the whole trial, all lambs received the same basal diet (composition shown in Table 1) at 07:00 and 17:00 every day in amounts exceeding the expected intake. Feed refusals were collected and weighed daily, and additional feed was supplemented as needed to maintain continuous feed availability. The amount of feed provided each day should be adjusted based on the previous day’s feed intake. Based on the recorded feed intake, the three CPP-C3M4 groups received CPP-C3M4 powder at doses of 0.5, 1.0, and 2.0 g per kg of basal diet, with the required amount calculated daily according to the previous day’s feed intake. The selected doses were determined based on preliminary dose-finding experiments. The powder was dissolved in 5 mL of sterile normal saline and infused through the duodenal fistula every day. The CON and ST groups received an equal volume of sterile normal saline via the same route. On the 33rd to 35th days of the experimental period, lambs in the ST group and three CPP-C3M4 groups were perfused with 5 mL of *S.* Typhimurium through the duodenal fistula at 08:00 every day (according to the pre-test results, the bacterial concentration was determined to be 1 × 10^13^ CFU/mL), while the CON group received sterile saline. All lambs were maintained under identical environmental conditions throughout the experiment. According to the nutrient requirements of meat-type sheep (NY/T 816-2021), the basal diet was formulated, as shown in Table 1.

### 2.4. Sample Collection

Lambs were fasted for 12 h prior to tissue collection. Body weights were measured, and liver tissues were weighed in order to calculate the organ index. For histological analysis, liver samples were maintained in 4% paraformaldehyde. The remaining tissue was aliquoted and stored at −80 °C.

### 2.5. Organ Index and Histopathological Analysis

The calculation of the liver organ index utilized the formula: Organ index (100%) = (Liver weight/Body weight) × 100.

Liver tissues were placed in a 4% paraformaldehyde solution and fixed at 4 °C for 24 h, followed by dehydrated steps using a series of ethanol solutions (70%, 80%, 95%, and 100%). The tissues were then cleared with xylene and embedded in paraffin, and 5-micrometer-thick sections were prepared. After hematoxylin-eosin (HE) staining, the sections were mounted with neutral resin.

### 2.6. Biochemical and Antioxidant Assays

Place liver samples in ice-cold normal saline solution (1:9, *w/v*) and homogenize. After centrifugation (3000 rpm, 10 min, 4 °C), the resulting supernatants were collected. Total protein content was quantified using a BCA assay kit (Beyotime Biotechnology, Shanghai, China).

Liver function was assessed through the measurement of specific biomarkers, including alanine aminotransferase (ALT), aspartate aminotransferase (AST), triglyceride (TG), and total cholesterol (TC) levels, which were determined using assay kits (Nanjing Jiancheng Bioengineering Institute, Nanjing, China).

To ascertain the antioxidant status of the liver tissues, several important indices were measured, namely glutathione peroxidase (GSH-Px), superoxide dismutase (SOD), malondialdehyde (MDA), and catalase (CAT). These parameters were quantified using kits from the same manufacturer, following the provided protocols.

### 2.7. Real-Time Quantitative PCR Analysis

The extraction of total RNA from liver tissues was performed using TRIzol reagent (Invitrogen, Beijing, China). RNA concentration and purity were assessed using a NanoDrop-2000 (Thermo Fisher Scientific, Waltham, MA, USA).

Take 1 μg of total RNA (with an OD260/280 ratio between 1.8 and 2.2) and use the HiScript III Reverse Transcriptase kit (Vazyme, Nanjing, China) to reverse transcribe it into complementary DNA (cDNA). Quantitative PCR (qPCR) was performed on a CFX96 system (Bio-Rad, Hercules, CA, USA) using SYBR Green qPCR mix (Innovagene, Changsha, China). The glyceraldehyde-3-phosphate dehydrogenase (GAPDH) gene was used as the internal control. Relative gene expression was calculated using the 2^−ΔΔCt^ method. Primer sequences are listed in Table 2.

### 2.8. Liver Transcriptome Analysis

#### 2.8.1. Library Construction and Sequencing

RNA extraction, quality assessment, and library construction were performed following the protocol described by Zhong et al. [31]. Total RNA was extracted from the tissue using TRIzol Reagent. Then RNA quality was determined by 5300 Bioanalyzer (Agilent Technologies, Santa Clara, CA, USA) and quantified using a NanoDrop-2000. Only high-quality RNA samples (OD260/280 = 1.8~2.2, OD260/230 ≥ 2.0, RQN ≥ 6.5) were used for library construction.

Transcriptome library preparation followed the Illumina TruSeq™ RNA Sample Prep Kit protocol (Illumina, San Diego, CA, USA). Briefly, oligo(dT)-conjugated magnetic beads were applied to isolate mRNA, which was then fragmented into pieces of 300 base pairs (bp). Using random primers and reverse transcriptase, first-strand cDNA was synthesized from the RNA fragmented and second-strand cDNA synthesis was subsequently performed. The obtained double-stranded cDNA underwent end repair, A-tailing, and adapter ligation. After purification and fragment selection, the ligation products were amplified by polymerase chain reaction (PCR) to construct the final library for sequencing. The total RNA extraction, library construction, sequencing, and bioinformatics analysis were carried out by Cosmos Wisdom Biotech Co., Ltd. in Hangzhou, China, using Illumina’s NovaSeq 6000 sequencing platform (Illumina, San Diego, CA, USA). Sequencing was performed in paired-end mode with a read length of 150 bp.

#### 2.8.2. Bioinformatics Data Processing

The raw sequencing data were processed using Fastp software (version 0.23.4) [32]. The Q20 and Q30 values for all samples were above 98.84% and 96.16%, respectively; the GC content ranged from 45.25% to 46.98%, and the unique mapping rate for each sample exceeded 90.66%, indicating that the data obtained were of high quality and suitable for subsequent analysis. Detailed quality control metrics are provided in Supplementary Table S1. Reads containing adapter sequences, reads with a N ratio > 10%, and low-quality reads were removed. Clean reads were aligned to reference genome (*Ovis aries* ARS-UI_Ramb_v2.0 release-112) using HISAT2 software (version 2.2.1) [33]. Subsequently, transcriptome assembly was performed using StringTie (version 2.2.1) [34], followed by quantification of gene and transcript expression levels in FPKM with RSEM (version 1.3.3) [35].

#### 2.8.3. Differential Gene Expression and Functional Analysis

Differential gene expression analysis was conducted using DESeq2 (version 1.42.0) [36] to compare the CON, ST, and CPP-C3M4-M groups. Differentially expressed genes (DEGs) were identified using a screening threshold of |log2 fold change| > 1 and *p*-value < 0.05. Functional analysis was carried out using goatools (version 1.4.4) for Gene Ontology (GO) and KOBAS (version 2.0) [37] for Kyoto Encyclopedia of Genes and Genomes (KEGG) pathway analysis.

#### 2.8.4. RT-qPCR Validation

Nine genes from the IL-17 signaling pathway identified by KEGG enrichment analysis, namely *IL-17A*, *IL-17RA*, *Act1*, *TRAF6*, *LCN2*, *HSP90B1*, *S100A8*, *S100A9*, and *CXCL10*, were selected for further validation. All RT-qPCR reactions followed the protocols described in Section 2.7, with primer sequences provided in Table 2.

### 2.9. Statistical Analysis

The experimental data were collated and calculated using Microsoft Excel 2021. Statistical analysis was performed with SPSS 27.0 software (IBM Corp., Armonk, NY, USA). All data were analyzed under a completely randomized single-factor design, with treatment group as a fixed effect. The statistical model was specified as:

*Y_ij_ = μ* + *T_i_* + *ε_ij_*

where *Y_i_*_j_ is the observed value of a given parameter for the j-th lamb in the i-th group, *μ* is the overall mean, *T_i_* is the fixed effect of the i-th treatment group (i = 1, 2, 3, 4, 5, representing the CON, ST, CPP-C3M4-L, CPP-C3M4-M, and CPP-C3M4-H groups, respectively), and *ε_ij_* is the random error assumed to be independently and normally distributed with homogeneous variance. The normality and homogeneity of variances were evaluated before analysis. For data meeting the assumptions, one-way ANOVA and Duncan’s method were used for multiple comparisons. For data that did not meet the above assumptions, Welch’s ANOVA was used for group comparisons, and Tamhane’s T2 test was used for multiple comparisons. Results are expressed as mean ± Standard Deviation (mean ± SD). *p* < 0.05 was considered statistically significant.

## 3. Results

### 3.1. Effects of CPP-C3M4 on Liver Weight and Liver Organ Index in Lambs

Compared with the CON group, *S.* Typhimurium infection significantly increased liver weight and liver organ index (*p* < 0.05; Figure 1). CPP-C3M4 pretreatment reversed these changes, as liver weights and organ indices in all three CPP-C3M4 pretreatment groups were significantly lower than those in the ST group (*p* < 0.05) and showed no significant difference from those in the CON group (*p* > 0.05).

### 3.2. Effects of CPP-C3M4 on Liver Histomorphology in Lambs

As shown in Figure 2, histological analysis revealed that the liver lobule structure was intact in the CON group, with no obvious pathological lesions. In contrast, the ST group exhibited severe histopathological damage, including diffuse hepatocellular edema, marked sinusoidal congestion with hemorrhage. Compared with the ST group, the CPP-C3M4-L group showed recovery from edema and congestion. The CPP-C3M4-M and CPP-C3M4-H groups presented nearly intact liver lobule structure, with significant reduction in hepatocellular edema, and no obvious sinusoidal congestion or hemorrhage in liver tissues.

### 3.3. Effects of CPP-C3M4 on Liver Biochemical and Antioxidant Parameters in Lambs

The effects of CPP-C3M4 pretreatment on liver function and lipid metabolism in lambs are shown in Figure 3A. *S.* Typhimurium infection significantly elevated ALT and AST activities compared with the CON group (*p* < 0.05). CPP-C3M4 pretreatment effectively overcame these changes, all CPP groups showed significantly decreased AST activity (*p* < 0.05), with the CPP-C3M4-M group exhibiting the lowest activity (*p* < 0.05), while ALT activity was significantly reduced in the CPP-C3M4-M and CPP-C3M4-H groups (*p* < 0.05), closer to CON levels (*p* > 0.05). Liver TG and TC contents did not differ between the ST and CON groups (*p* > 0.05). TG content was significantly reduced in the CPP-C3M4-L and CPP-C3M4-M groups (*p* < 0.05), but TC content showed no significant changes across all CPP groups (*p* > 0.05).

Regarding oxidative stress (Figure 3B), CAT and GSH-Px activities were significantly decreased in the ST group compared with the CON group (*p* < 0.05), while SOD activity and MDA content showed no significant differences (*p* > 0.05). CPP-C3M4 pretreatment effectively counteracted these impairments, all CPP doses significantly increased GSH-Px activity to CON levels (*p* < 0.05); only the medium dose significantly restored CAT activity (*p* < 0.05); the medium and high doses significantly increased SOD activity (*p* < 0.05); MDA content showed no significant variation across all groups (*p* > 0.05).

### 3.4. Effects of CPP-C3M4 on Liver Inflammatory Mediator mRNA Expression in Lambs

Figure 4 shows the expression levels of mRNA for inflammatory mediators in lamb liver tissue. *Salmonella* infection caused a significant upregulation in the mRNA expression of *IL-2*, *IL-6*, *IL-1β*, *TNF-α*, *TLR2*, and *TLR9* compared with the CON group (*p* < 0.05). CPP-C3M4 pretreatment alleviated this inflammatory response. Compared with the ST group, all CPP-C3M4 pretreatment groups showed significantly reduced mRNA levels of *IL-2*, *IL-6*, *TNF-α*, *TLR2*, and *TLR9* (*p* < 0.05), while *IL-1β* expression was significantly decreased only in the CPP-C3M4-M and CPP-C3M4-H groups (*p* < 0.05).

### 3.5. Liver Transcriptomics Analysis

The protective effects of three doses (low, medium, and high) of CPP-C3M4 against *Salmonella*-induced liver injury were evaluated using a variety of indicators, including liver function parameters, antioxidant capacity, and inflammatory cytokine expression levels. Among these, the medium dose (CPP-C3M4-M) exhibited the most consistent and balanced protective effects, and was therefore selected for transcriptomic analysis. To elucidate the underlying liver protective mechanism of CPP-C3M4 pretreatment, liver tissues from the CON, ST, and CPP-C3M4-M groups were subjected to transcriptome sequencing.

#### 3.5.1. Differential Gene Expression Analysis

The DEGs were identified using DESeq2 according to the criteria of *p* < 0.05 and |log2(fold change)| > 1. The volcano plot of ST group vs. CON group showed that a total of 1321 genes were differentially expressed, with 623 genes upregulated and 698 genes downregulated (Figure 5A). Comparatively, 401 DEGs were identified in CPP-C3M4-M group vs. ST group, including 152 upregulated genes and 249 downregulated genes (Figure 5B).

#### 3.5.2. Gene Ontology Enrichment Analysis

In the ST vs. CON group (Figure 6A), GO enrichment analysis showed that among the top 10 enriched terms in the Biological Process (BP) category, only the small molecule biosynthetic process was significantly enriched (adjusted *p* < 0.05). In the Cellular Component (CC) category, only extracellular region was significantly enriched (adjusted *p* < 0.05) among the top 10 terms. In contrast, all of the top 10 enriched terms in the Molecular Function (MF) category reached significant levels (adjusted *p* < 0.05), mainly including oxidoreductase activity, iron ion binding, transition metal ion binding, electron transfer activity, and transmembrane transporter activity.

In the ST vs. CPP-C3M4-M group (Figure 6B), GO enrichment analysis indicated that the significantly enriched BP terms were lipid metabolic process and regulation of hormone levels (adjusted *p* < 0.05). Four CC terms were significantly enriched (adjusted *p* < 0.05), namely nucleosome, chromatin, chromosome, and protein-DNA complex. In the MF category, only the structural constituent of chromatin and protein heterodimerization activity showed significant enrichment (adjusted *p* < 0.05), whereas other terms such as oxidoreductase activity and iron ion binding did not reach statistical significance (adjusted *p* > 0.05).

#### 3.5.3. KEGG Enrichment Analysis

To elucidate the transcriptional mechanisms of *Salmonella*-induced liver injury and the protective effect of CPP-C3M4-M, KEGG pathway enrichment analysis was performed on DEGs in each comparison group (Figure 7). Significant enrichment was defined as adjusted *p* < 0.05.

In the ST vs. CON comparison, upregulated DEGs (Figure 7A) were significantly enriched in inflammatory and immune pathways, including neutrophil extracellular trap formation, NOD-like receptor signaling pathway, IL-17 signaling pathway, TNF signaling pathway, NF-κB signaling pathway and Toll-like receptor signaling pathway. Additional significantly enriched immune-related pathways included cytokine-cytokine receptor interaction and phagosome. Downregulated DEGs (Figure 7B) were mainly enriched in metabolic pathways, including steroid hormone biosynthesis, retinol metabolism, carbon metabolism, and xenobiotic metabolism (e.g., drug metabolism–cytochrome P450, metabolism of xenobiotics by cytochrome P450), as well as chemical carcinogenesis-reactive oxygen species (related to oxidative stress).

In the ST vs. CPP-C3M4-M comparison, the classical inflammatory pathways (IL-17, TNF, NF-κB, and NOD-like receptor signaling pathways) that were significantly enriched in ST vs. CON were no longer significantly enriched. Instead, upregulated DEGs were enriched in systemic lupus erythematosus, neutrophil extracellular trap formation, and alcoholism pathways (Figure 7C). Downregulated DEGs were mainly associated with metabolic recovery, including steroid biosynthesis, carbon metabolism, and retinol metabolism, as well as pantothenate and CoA biosynthesis and terpenoid backbone biosynthesis (Figure 7D).

#### 3.5.4. RT-qPCR Analysis of IL-17 Signaling Pathway-Associated Genes

Nine genes of the IL-17 signaling pathway (*IL-17A*, *IL-17RA*, *Act1*, *TRAF6*, *HSP90B1*, *LCN2*, *S100A8*, *S100A9*, and *CXCL10*) were selected for RT-qPCR (Figure 8). All target genes were significantly upregulated in the ST group compared with the CON group (*p* < 0.05), indicating that the IL-17 signaling pathway was strongly activated following *Salmonella* infection. Pretreatment with CPP-C3M4-M markedly attenuated this induction, with expression levels significantly lower than those in the ST group (*p* < 0.05).

## 4. Discussion

This study investigated the protective effect of CPP-C3M4 on *Salmonella*-induced liver injury in lambs from multiple perspectives, including tissue morphology, liver function, oxidative stress, inflammatory factors, and transcriptomic analysis, and revealed its multi-target regulatory pathways.

*S.* Typhimurium infection significantly increased the weight and organ index of lamb liver (Figure 1). Histopathological examination revealed obvious inflammatory cell infiltration and tissue edema, which was consistent with the observations in mouse models [38,39,40]. CPP-C3M4 pretreatment significantly reduced liver weight and liver organ index, restored histopathological morphology, and decreased the elevated AST and ALT activities in the infected group [41], collectively indicating that the antimicrobial peptide ameliorated *Salmonella*-induced structural damage and functional impairment of the liver. The reduction in AST and ALT, which are enzymes normally confined within hepatocytes, reflects the preservation of hepatocellular membrane integrity. The histopathological structure was restored, as evidenced by a reduction in inflammatory cell infiltration and tissue edema, indicating that the liver inflammatory response had been alleviated and tissue homeostasis improved. This hepatoprotective effect is consistent with the results reported in previous studies on other antimicrobial peptides. For instance, antimicrobial peptide YD has been shown to reduce serum transaminase activity and improve histopathological damage in mice with carbon tetrachloride-induced liver fibrosis [42]; the cationic antimicrobial peptide cathelicidin-PY can alleviate the extent of hepatocyte necrosis and inflammatory cell infiltration in an acute liver failure model [43].

As a typical intracellular pathogen, *Salmonella* releases pathogen-associated molecular patterns (PAMPs), which are recognized by Toll-like receptors (TLRs) expressed on the surface of liver immune cells, including Kupffer cells and dendritic cells [44]. The results of this study show that the mRNA levels of *TLR2* and *TLR9* were significantly upregulated in the infected group, and the expression levels of downstream pro-inflammatory cytokines, such as *IL-6*, *TNF-α*, *IL-1β* and *IL-2*, were also markedly elevated, reflecting a stepwise amplification process from receptor recognition to the production of effector molecules. The mechanistic significance of TLR pathway activation for hepatotoxicity is that sustained TLR2/9 signaling in Kupffer cells promotes the release of pro-inflammatory mediators that directly induce hepatocyte apoptosis and amplify local tissue damage through neutrophil recruitment and extravasation [45]. CPP-C3M4 pretreatment effectively inhibited the expression of *TLR2/9* and various inflammatory factors, thereby interrupting this inflammatory cascade at its initiation and reducing the inflammatory burden on hepatocytes. Transcriptomic KEGG analysis further confirmed this effect. The inflammatory pathways significantly enriched in the ST group, including IL-17, TNF, NF-κB, and NOD-like receptor pathways, were no longer significantly enriched following antimicrobial peptide CPP-C3M4 intervention. Among these, the IL-17 signaling pathway emerged as a key axis regulated by CPP-C3M4. Combined transcriptomic analysis and qPCR results showed that multiple critical genes in this pathway (such as *IL-17A*, *IL-17RA*, *Act1*, *TRAF6*, *LCN2*, *S100A8*, *S100A9*, and *CXCL10*) were markedly upregulated after infection but downregulated following CPP-C3M4 pretreatment (Figure 8). These results indicate that CPP-C3M4 attenuates *Salmonella*-induced liver inflammation through the downregulation of TLR2/9-mediated inflammatory signaling and the suppression of key pro-inflammatory cytokines and pathways, including the IL-17 axis. The concurrent downregulation of inflammation-associated molecules such as *S100A8/A9* and *LCN2* further supports the anti-inflammatory effect of CPP-C3M4. Previous studies have confirmed that antimicrobial peptides (e.g., NK-lysin [46], [K4K15]CZS-1 [47], HJH-3 [48]) can disrupt *Salmonella* cell membrane integrity, thereby reducing the release of PAMPs. Although this study did not directly assess bacterial membrane integrity, the observed downregulation of *TLR2* and *TLR9* mRNA expression suggests that CPP-C3M4 may reduce the release of PAMP through a similar mechanism, consequently inhibiting the activation of the Toll-like receptor signaling pathway at its source. Further research is needed to elucidate its potential mechanism of action.

In the ST group, the activities of CAT and GSH-Px in lamb liver tissue were significantly reduced. This damage impaired the organism’s ability to clear hydrogen peroxide (H_2_O_2_) and lipid peroxides, leading to the accumulation of reactive oxygen species (ROS) [49]. The functional consequence of this impaired antioxidant system is the inability to neutralize ROS, which then attack the polyunsaturated fatty acids in liver cell membranes, triggering lipid peroxidation and disrupting the integrity of the cell membranes. MDA, as the main end product of lipid peroxidation, can interact with cell membranes and proteins, exacerbating tissue damage and forming a vicious cycle of oxidative stress [50]. Consistent with our findings, Li et al. [51] observed that *S.* Typhimurium infection significantly reduced GSH-Px and SOD activities, while elevating MDA levels in mouse liver. Their study further confirmed that *Salmonella* infection exacerbates liver oxidative damage by disrupting the antioxidant system. Administration of CPP-C3M4 via a duodenal fistula alleviated the *Salmonella*-induced decline in liver antioxidant enzyme activity. Among the tested doses, 1.0 g/kg CPP-C3M4 exerted the most potent antioxidative effects, as evidenced by enhanced activities of CAT, GSH-Px, and SOD in the liver. The restoration of these antioxidant enzymes by CPP-C3M4 implies a reconstituted capacity to scavenge ROS, thereby breaking the vicious cycle of oxidative damage and contributing to the recovery of hepatocyte function. GO analysis revealed that oxidoreductase activity, iron ion binding, electron transfer activity, and other oxidative stress-related terms significantly enriched in the ST group were no longer significantly enriched following CPP-C3M4 intervention. Meanwhile, KEGG analysis showed that the “chemical carcinogenesis-ROS” pathway, which was enriched in the ST group, was no longer significant in the CPP-C3M4 group. This consistency between biochemical enzyme activities and transcriptomic pathway alterations provides multi-level evidence for the antioxidant efficacy of CPP-C3M4, ranging from functional proteins to gene networks.

The antioxidant effects of antimicrobial peptides can be attributed to multiple mechanisms. Some antimicrobial peptides, such as LL-37 can directly neutralize H_2_O_2_ and superoxide anions [52]. Others indirectly enhance antioxidant enzyme activity by regulating endogenous antioxidant pathways; for example, MS15 activates the Nrf2-HO-1 signaling pathway to upregulate the expression of CAT, GSH-Px, and SOD in macrophages [53]. Although the specific antioxidant mechanism of CPP-C3M4 was not fully elucidated in this study, these findings provide a basis for future investigations. It should be noted that no significant change in MDA level was observed in this study, which may reflect the relatively short intervention period or the specific infection model employed. Nevertheless, the marked increase in the activities of three key antioxidant enzymes sufficiently demonstrates the antioxidant capacity of CPP-C3M4.

Transcriptomic KEGG analysis showed that immune/inflammation-related pathways were upregulated in the ST group, whereas metabolic pathways were downregulated. This opposite enrichment pattern suggests that *Salmonella* infection induces reprogramming of the liver transcriptome. Specifically, this reprogramming is characterized by transcriptional activation of immune defense genes accompanied by transcriptional suppression of metabolic homeostasis genes. Chen et al. [54] observed a similar transcriptomic remodeling phenomenon in the livers of broiler chickens infected with avian pathogenic *Escherichia coli*.

In the present study, *Salmonella* infection did not significantly alter liver TG or TC contents. This finding indicates that inflammation and oxidative stress are the primary manifestations of liver injury during the early stage of acute infection, rather than lipid metabolism disorders [55].

In lambs pretreated with CPP-C3M4, pathways involved in carbon metabolism, steroid hormone biosynthesis, retinol metabolism, and glyoxylate and dicarboxylate metabolism were significantly enriched in the transcriptome, indicating that CPP-C3M4 pretreatment partially reversed the infection-induced metabolic dysregulation. The recovery of these metabolic pathways may contribute to the improved liver function observed in the CPP-C3M4-treated group.

Although the present study focused on hepatic protection, the anti-inflammatory and immunomodulatory effects of CPP-C3M4 observed in the liver, particularly the suppression of TLR2/9 signaling and downregulation of IL-6, TNF-α, and IL-1β, may also be relevant to intestinal protection [56]. These same inflammatory mediators are central to the pathogenesis of *Salmonella* enteritis [57,58], and antimicrobial peptides have been reported to attenuate intestinal inflammation by reducing bacterial translocation and modulating mucosal immune responses. Although the present study lacked direct evaluation of intestinal tissue, the mechanistic insights obtained lay the groundwork for future investigations into the protective effects of CPP-C3M4 against *Salmonella* enteritis in lambs, which is a clinically prevalent and challenging condition.

The growing crisis of antibiotic resistance has limited treatment options for *Salmonella* infections in livestock, creating an urgent need to develop alternative treatment strategies. Antimicrobial peptides exert their antibacterial effects by disrupting the cell membrane; compared to traditional antibiotics, this mechanism of action is less likely to induce bacterial resistance. In this study, a standard reference strain was used, and the direct activity of CPP-C3M4 against multidrug-resistant clinical isolates was not evaluated. Furthermore, this study did not measure the bacterial count in liver tissue; hence, it was unable to directly assess the association between bacterial load in the liver and tissue damage. Moreover, *Salmonella*-induced liver injury is not a common clinical manifestation in lambs; the results of this study can only be directly applied to clinical translational research on liver-related complications, and their applicability is limited. Nevertheless, our histopathological and biochemical findings strongly corroborated the transcriptomic data, providing multi-level evidence for the hepatoprotective effect. Future studies incorporating fresh tissue sampling for bacteriological analysis and efficacy testing against resistant strains, as well as evaluation of protection against *Salmonella* enteritis, will further clarify the translational potential of CPP-C3M4.

## 5. Conclusions

CPP-C3M4 attenuates *Salmonella*-induced liver damage in lambs through multiple mechanisms, thereby exerting a protective effect. These mechanisms include suppressing excessive inflammatory responses, enhancing the body’s own antioxidant capacity, and partially reversing metabolic suppression. The protective effect is consistently supported by multiple independent indicators, such as tissue morphology, liver function, oxidative stress, inflammatory factors, and transcriptomic analysis. This study provides strong experimental evidence for CPP-C3M4 as a potential candidate for preventing *Salmonella*-induced liver injury in lambs. Future studies are warranted to evaluate the protective efficacy of CPP-C3M4 against *Salmonella* enteritis, which represents a more prevalent clinical challenge in lambs. In addition, investigations using multidrug-resistant clinical isolates and incorporating fresh tissue sampling for bacteriological analysis would further clarify the translational potential of CPP-C3M4.

## Figures and Tables

**Figure 1:**
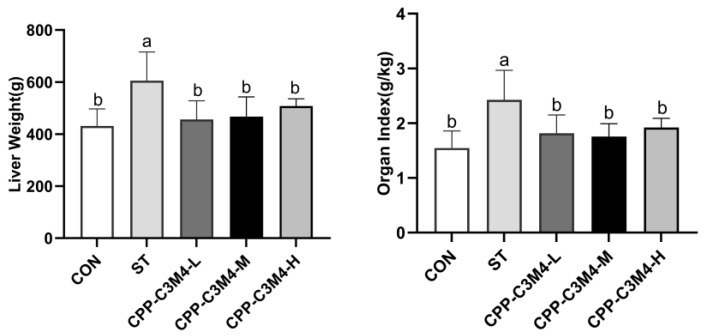
Liver weight and liver organ index in lambs. The different letters above the bar charts (a, b) indicate significant differences between groups (*p* < 0.05).

**Figure 2:**
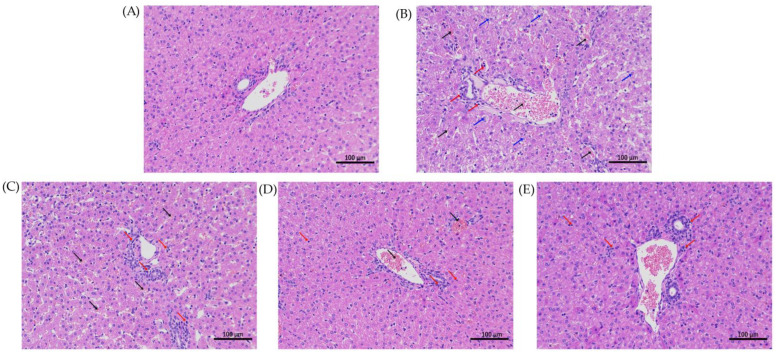
H&E-stained liver tissue sections in lambs (200× magnification; scale bar = 100 μm). Arrows indicate specific pathological features: black arrows, sinusoidal congestion and hemorrhage; red arrows, inflammatory cell infiltration; blue arrows, hepatocyte degeneration. (**A**) CON group: normal liver tissue structure. (**B**) ST group: severe sinusoidal congestion (black arrows), extensive inflammatory cell infiltration (red arrows), and marked hepatocyte degeneration (blue arrows). (**C**) CPP-C3M4-L group: showed partial improvement, with reduced sinusoidal congestion (black arrows), residual inflammatory cell infiltration (red arrows), and slight hepatocyte degeneration (blue arrows). (**D**,**E**) CPP-C3M4-M and CPP-C3M4-H groups: liver tissue structure was well restored with marked resolution of pathological changes.

**Figure 3:**
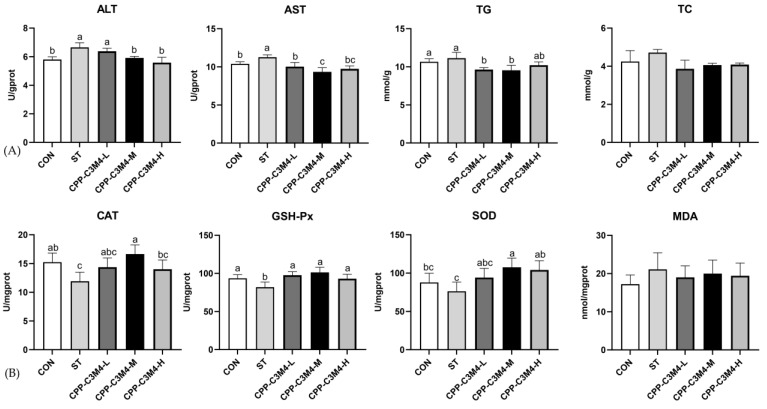
Effects of CPP-C3M4 on liver biochemical and antioxidant parameters in lambs. (**A**) Liver biochemical indicators; (**B**) Liver antioxidant status. Different letters (a, b, c) above the bars indicate significant differences between groups (*p* < 0.05).

**Figure 4:**
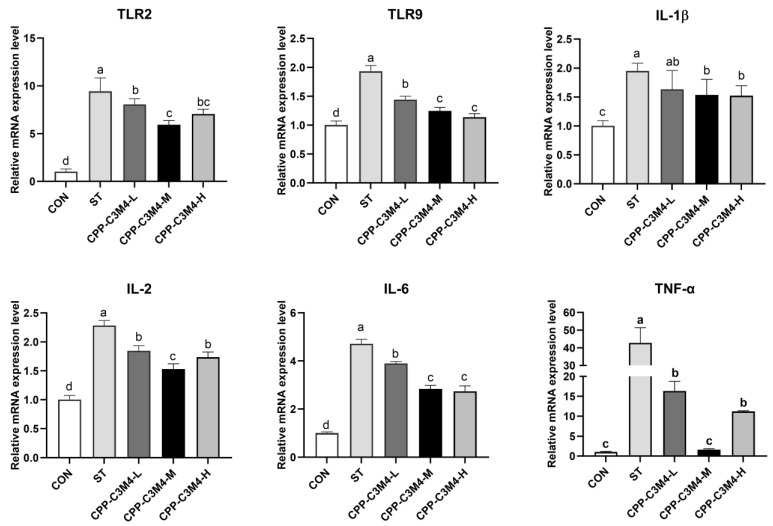
Liver inflammatory mediator mRNA expression levels in lambs. Different letters (a, b, c, d) above the bars indicate significant differences between groups (*p* < 0.05).

**Figure 5:**
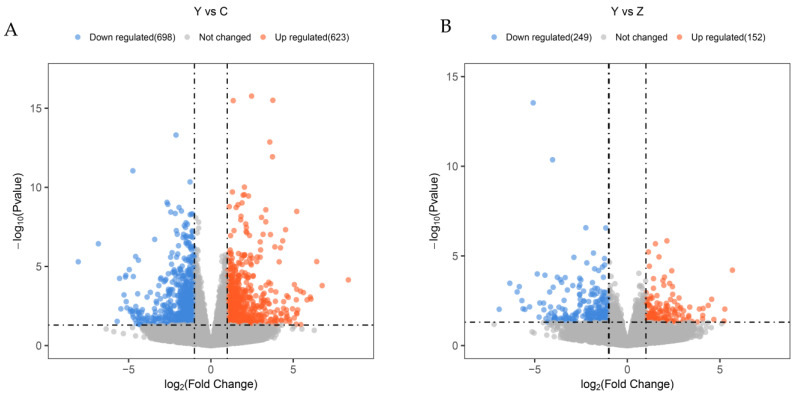
Volcano plot of differential gene expression analysis in lamb liver tissue: (**A**) ST vs. CON; (**B**) ST vs. CPP-C3M4-M. Red and blue dots indicate upregulated and downregulated genes, respectively.

**Figure 6:**
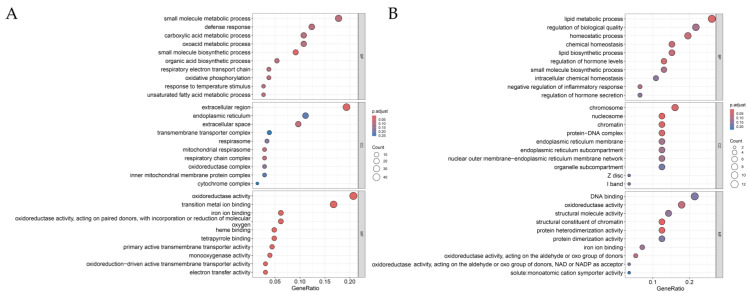
GO enrichment analysis of DEGs in lamb liver. (**A**) ST vs. CON (**B**) ST vs. CPP-C3M4-M.

**Figure 7:**
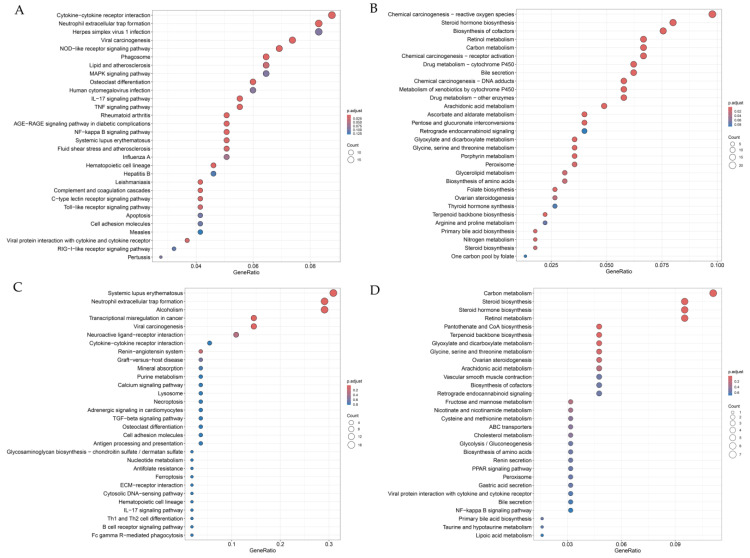
KEGG pathway enrichment analysis of DEGs in lamb liver. (**A**) ST vs. CON (upregulated). (**B**) ST vs. CON (downregulated). (**C**) ST vs. CPP-C3M4-M (upregulated). (**D**) ST vs. CPP-C3M4-M (downregulated). The *x*-axis represents GeneRatio, and the *y*-axis shows KEGG pathways. Dot size indicates gene count, and dot color indicates *p*.adjust.

**Figure 8:**
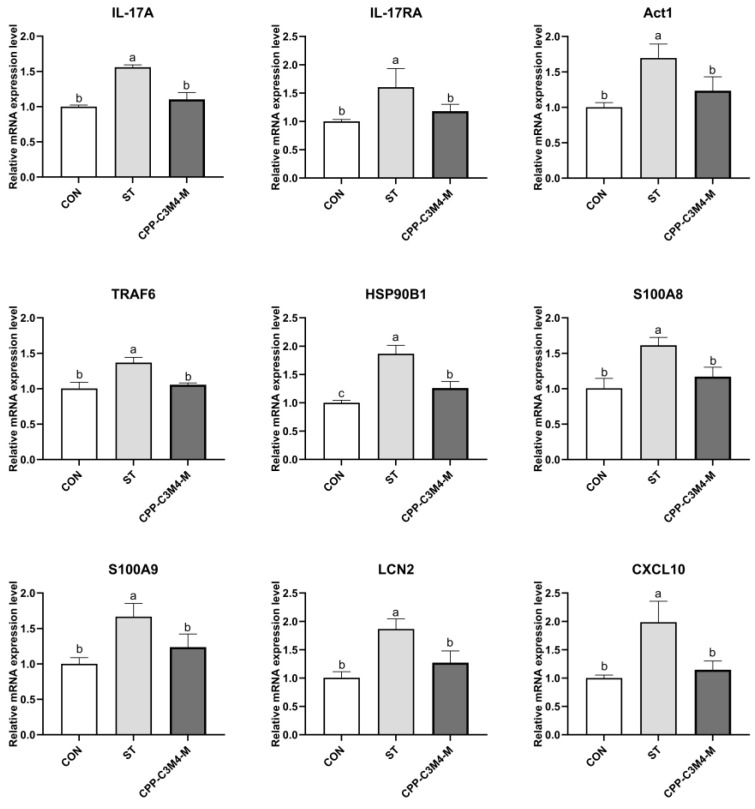
Relative expression levels of DEGs in the IL-17 signaling pathway in lamb liver among different groups. Letters (a, b and c) indicate significant differences (*p* < 0.05) among the groups.

**Table 1:** Composition and nutrient levels of the basal diet (air-dry basis).

Ingredients	Content, %	Nutrient Levels ^3^	Content, %
*Leymus chinensis*	20.00	DM	90.47
Alfalfa	10.00	ME(MJ/kg)	10.80
Corn	49.00	CP	15.99
Soybean meal	11.07	MP	7.36
Corn protein meal	1.00	DCP	11.92
Peanut meal	3.00	EE	3.90
Cottonseed meal	1.00	NDF	27.19
Rapeseed meal	1.00	ADF	14.30
Palm fat powder	1.00	Ca	0.93
CaHPO_4_	1.20	P	0.52
Limestone	0.60	Ash	4.23
NaCl	0.33		
Methionine	0.10		
Vitamin premix ^1^	0.20		
Trace element premix ^2^	0.50		
Total	100.00		

^1^ Vitamin premix per kg of diet: VA 11,000 IU, VD 5000 IU, VE 50 mg, VB_1_ 25 mg, VB_2_ 50 mg, VB_6_ 22 mg, VB_12_ 0.3 mg, VB_5_ 22 mg. ^2^ Trace mineral premix per kg of diet: Fe 75 mg, Cu 14 mg, Mn 60 mg, Zn 85 mg; I 0.5 mg, Se 0.3 mg; Co 0.3 mg. ^3^ ME was a calculated value; all other nutrient values were determined experimentally.

**Table 2:** Primer sequences used in this study.

Gene	Sequence (5′-3′)	Accession Number
*IL-2*	F: ACACCAGAGAGATCAAGGATTCAATG R: CCTTTACTGTCGCATCATCATATTCAC	NM_001009806.1
*IL-6*	F: TCGCAGGTCTAATAACCACTCCAG R: CAAGGCTTCTCAGGATGATGATAACC	NM_001009392.1
*IL-1β*	F: GACTTCAGAATGGAAACCCTCTCTC R: AGGCTGCGGCTGGTGTTC	NM_001009465.2
*TLR2*	F: TCTTCCTGTTGCTCCTGCTCAC R: CCTTCCTGGGCTTCCTCTTGG	XM_042234035.2
*TLR4*	F: GCGGAATGAACTGGTAAAGAACTTG R: GGATGATATTGGCGGCGATGG	NM_001135930.1
*TNF-α*	F: TCTCTCATACACCCTGCCACAAG R: CTCCGCCAGCTCCACATCC	NM_001024860.1
*IL-17A*	F: CTGTGATCTGGGAGGCCAAGTG R: AGGATCTCTTGCTGGATGGTGAC	XM_004018887.6
*IL-17RA*	F: CCTTGGTCCTGGCACTCCTG R: AGGCTGGTTCGTCGTTCTCC	XM_027968052.2
*Act1*	F: TTGGTCTGGTGCCTGGTCTG R: GTCTGGAACAACTGCTCATCGC	XM_002369622.2
*TRAF6*	F: GTTGGCGGTTGTATCGGTGAC R: GGCGGACAGCGACCCTTC	XM_015101111.3
*LCN2*	F: ACAAACAAACACACAAGGGAGAGAG R: ATGGCAGCGGCAGCAGAG	XM_042245652.2
*HSP90B1*	F: AGCGGAGACAGACCAACTAGAAG R: GCAGGCAGGTCGGTGAGC	XM_060413139.1
*S100A8*	F: ATGCTGACGGATCTGGAGAGTG R: GTCTCTAACAGTCTCTTCAAGTCATCC	XM_004002523.4
*S100A9*	F: TCAAGAAGCAGCATAAGGATGAAGAG R: GCCACCAGCATAATGAACTCCTC	XM_012181723.3
*CXCL10*	F: CGAACACAGAAAGAGGCATAATCAC R: CACGAACAATTAGGGCTTGACATATAC	NM_001009191.1
*GAPDH*	F: GGCCTCCAAGGAGTAAGGTC R: TCTCTTCCTCTCGTGCTCCT	XM_060411595.1

## Data Availability

The original contributions presented in this study are included in the article. Further inquiries can be directed to the corresponding author.

## Supplementary Materials

The following supporting information can be downloaded at: https://www.mdpi.com/article/10.3390/ani16152290/s1, Table S1. Quality analysis of transcriptome sequencing data.
