# Supplementary material for: Antimicrobial Peptide CPP-C3M4 Attenuates Salmonella-Induced Liver Inflammation and Oxidative Stress in Lambs
Source: Animals (Basel). 2026 Jul 23;16(15):2290. doi: 10.3390/ani16152290 (PMC13463587; doi:10.3390/ani16152290)
Supplement: Supplementary file 1 [file animals-16-02290-s001.zip › animals-4367331-supplementary.pdf]

Supplementary Table S1. Quality analysis of transcriptome sequencing data

Groups[1]	Raw reads	Clean reads	Q20 (%)	Q30 (%)	GC content (%)	Uniquely mapped	Mapping rate (%)
C1	41,940,532	41,704,320	99.01	96.73	45.42	38,217,392	91.64
C2	43,905,490	43,665,340	99.02	96.72	45.96	39,944,854	91.48
C3	43,379,376	43,160,704	99.05	96.85	45.25	39,523,056	91.57
Y1	48,270,130	47,987,880	99.02	96.78	46.98	43,758,786	91.19
Y2	40,489,962	40,255,452	98.98	96.62	46.40	36,495,082	90.66
Y3	40,778,970	40,519,128	98.96	96.58	46.36	36,911,534	91.10
Z1	40,593,342	40,372,162	98.99	96.64	46.03	37,066,700	91.81
Z2	39,826,088	39,591,900	98.99	96.66	45.79	36,334,770	91.77
Z3	42,540,276	42,259,360	98.84	96.16	46.60	38,540,010	91.20

[1] C, Y, and Z denote the CON, ST, and CPP-C3M4-M groups, respectively; numbers 1–3 represent three biological replicates per group.
